# Clinical characteristics and rehabilitation potential in children with cerebral palsy based on MRI classification system

**DOI:** 10.3389/fped.2024.1382172

**Published:** 2024-04-25

**Authors:** Jie Yang, Congjie Chen, Ningning Chen, Helin Zheng, Yuxia Chen, Xiaoli Li, Qingxia Jia, Tingsong Li

**Affiliations:** ^1^ Department of Rehabilitation, Children’s Hospital of Chongqing Medical University (CHCMU), Chongqing, China; ^2^ National Clinical Research Center for Child Health and Disorders, Chongqing, China; ^3^ Ministry of Education Key Laboratory of Child Development and Disorders, Chongqing, China; ^4^ Chongqing Key Laboratory of Pediatrics, Chongqing, China; ^5^ Department of Radiology, CHCMU, Chongqing, China

**Keywords:** department of radiology, CHCMU cerebral palsy, MRI classification system, clinical characteristics, rehabilitation potential

## Abstract

**Background:**

The correlation of clinical characteristics of cerebral palsy (CP) and the magnetic resonance imaging classification system (MRICS) for (CP) is inconsistent. Specifically, the variance in rehabilitation potential across MRICS remains underexplored.

**Aims:**

To investigate the clinical characteristics and potential for rehabilitation in children with CP based on MRICS.

**Materials and methods:**

Children with CP admitted to the Department of Rehabilitation, Children's Hospital of Chongqing Medical University between 2017 and 2021 were included in the study. Qualified cases underwent a follow-up period of at least one year. The clinical characteristics of CP among different MRICS were analyzed, then the rehabilitation potential was explored by a retrospective cohort study.

**Results:**

Among the 384 initially enrolled children, the male-to-female ratio was 2.3:1, and the median age of diagnosis was 6.5 months (interquartile range: 4–12). The most prevalent MRICS categorization was predominant white matter injury (40.6%), followed by miscellaneous (29.2%) and predominant gray matter injury (15.6%). For the predominant white matter injury and miscellaneous categories, spastic diplegia emerged as the leading subtype of CP, with incidences of 59.6% and 36.6%, respectively, while mixed CP (36.7%) was the most common type in children with predominant gray matter. Notably, 76.4% of children with predominant white matter injury were classified as levels I–III on the gross motor function classification system (GMFCS), indicating significantly less severity than other groups (*χ*2^ ^= 12.438, *p *= 0.013). No significant difference across MRICS categories was observed for the manual ability classification system (MACS) (*H* = 8.176, *p = *0.085). Rehabilitation potential regarding fine motor function and adaptability based on Gesell assessment was dependent on MRICS over the follow-up period. Children with normal MRI scans exhibited superior rehabilitation outcomes. Commencing rehabilitation at an earlier stage produced consistent and beneficial results in terms of fine motor function and adaptability across all MRICS categories. Moreover, participants below 2 years of age demonstrated enhanced rehabilitation potential regarding fine motor outcomes and adaptability within the MRICS framework.

**Conclusion:**

MRICS displayed a significant association with clinical characteristics and rehabilitation efficacy in children with CP.

## 1 Introduction

Cerebral palsy (CP) refers to a group of enduring disorders resulting in activity limitations due to non-progressive lesions in the developing brain (1). It is the primary cause of childhood physical disability, affecting between 1.5 to 3.0 children per 1,000 live births (2). While CP is primarily diagnosed based on neurological symptoms and motor impairment, brain anomalies detected through magnetic resonance imaging (MRI) can shed light on the structural-functional relationship and exact timing of the causative injury.

In 2016, the Surveillance of Cerebral Palsy in Europe (SCPE) group introduced the MRI classification system (MRICS), which has been validated as both applicable and reliable for communication across various CP registers (3). Subsequently, multiple studies have explored the relationship between MRICS patterns and CP type, functional impairment, and comorbidities, yielding inconsistent conclusions (4–8). For example, most studies have identified white matter injury as the most common type, whereas in Africa it was grey matter injury because the main etiology of CP there were birth asphyxia and bilirubin encephalopathy (8). Unilateral spastic paralysis was associated with grey matter injury, but it was more common in white matter injury in Horber's study (4). Some studies have found that children with white matter injury and normal MRI finding were more mild, while Nagt's study found that grey matter injury also had better motor ability and intelligence (6). Nagy's study showed that miscellaneous group combined with epilepsy was more common (6), while Lovric's study suggested that it was the rarest (7).

Rehabilitation potential refers to the additional improvement of a patient with rehabilitation interventions over time from clinicians' prediction (9). Deciding the rehabilitation potential of cases with brain injury is challenging due to the various inflicted disorder and assessors (10). The MRICS categories not only denote patterns of brain development and etiology, but also indicate the timing of brain injury (3). For instance, injuries to the periventricular white matter predominantly occur during the third trimester, a stage characterized by prominent synapse formation, dendritic growth, and myelination onset. Notably, the alignment of injury timing with gestational age and specific structural attributes, including the configuration of corticospinal tract projections, is correlated with motor outcomes (7). Therefore, it is reasonable to hypothesize that motor function outcome and rehabilitation potential may differ across MRICS patterns. To date, however, few studies have explored this topic.

In the present study, we conducted a cross-sectional survey to describe the relationship between MRICS patterns, motor function, and neurodevelopmental milestones. We also analyzed rehabilitation potential based on a retrospective cohort study to elucidate the clinical relevance of MRICS.

## 2 Materials and methods

### 2.1 Participants

Children with CP admitted to the Department of Rehabilitation, Children's Hospital of Chongqing Medical University between January 2017 and December 2021 were enrolled in the study. In our study, the diagnose criteria were as follows (1): (1) a permanent disorder of movement and/or posture and of motor function; (2) It is due to a non-progressive interference, lesion, or abnormality occurred in the immature brain. The diagnosis of CP before the age of 2 years was preliminary and follow-up was needed for them. The inclusion criteria were as follows: adherence to the CP definition (1); complete brain MRI data; initial assessments based on gross motor function measure-88 (GMFM-88), fine motor function measure (FMFM), and Gesell data prior to rehabilitation. The exclusion criteria were as follows: disease other than CP established during rehabilitation; incomplete MRI data; and incomplete GMFM, FMFM, and Gesell assessments. Only subjects with at least 12 consecutive months of at-home and in-hospital rehabilitation were included for potential analysis. Systematic rehabilitation by professional physiotherapists covered conventional functional rehabilitation, functional task-oriented approaches, physical and sports activities, occupational therapies, technology assisted rehabilitation and education interventions based on the individual conditions (11). The average rehabilitation time was more than four hours per day, whether at home or in hospital. Changes in scores pertaining to motor function and neurodevelopmental milestones within the first six months and 7–12 months post-rehabilitation were compared. The former and latter periods were regarded as the early and late treatment periods, respectively. For cases included in rehabilitation capability analysis, a minimum of six months of task-specific motor training for all subtypes, constraint-induced movement therapy for hemiplegia, and cognitive intervention (administered in cases of identified cognitive impairment) (12) were initiated post CP diagnosis.

### 2.2 Data collection

In the present study, various data were extracted from medical records, including: (1) demographic details, such as sex, age at diagnosis, admission time, gestational age, and birth weight; (2) MRI findings of the brain; and (3) clinical data, including diagnosis, CP subtype, and scores related to gross motor function classification system (GMFCS), manual ability classification system (MACS), Viking speech scale (VSS), GMFM-88, FMFM, Gesell scale, and Wechsler intelligence scale (WISC).

All MRI findings were reclassified according to MRICS by a radioneurologist. Patterns were categorized as (A) maldevelopments, (B) predominant white matter injury, (C) predominant gray matter injury, (D) miscellaneous, or (E) normal findings (3).

The CP subtype was classified into spastic, ataxia, dyskinetic, and mixed types. Spastic CP was divided into diplegia, hemiplegia, or tetraplegia CP (13).

Evaluations were conducted using the Gesell scale, GMFCS, MACS, VSS, and WISC to measure developmental milestones, gross/fine motor skills, and language development individually. Within the Gesell assessment, the fine motor capability of children with hemiplegia was determined by the average development quotient (DQ) of bilateral fine motor skills. For WISC, scores falling below the established threshold were documented as the threshold value.

Rehabilitation potential in gross and fine motor skills was evaluated using GMFM and FMFM, represented by the change in score compared to initial assessment. The Gesell scale was used to evaluate developmental domains comprising sociability, adaptability, language, and gross and fine motor milestones. Based on age at diagnosis, the cohort was divided into two groups: ≤2 years and >2 years.

### 2.3 Statistical analysis

SPSS v25.0 and RStudio were used for statistical analysis. Non-normally distributed data were presented as medians and interquartile range (IQR), and rank variables were compared using the Kruskal-Wallis test. Data showing normal distribution were expressed as means ± standard deviation (SD), and differences were compared using *t*-test or analysis of variance (ANOVA). Categorical variables were summarized as frequencies and percentages, and compared using the *χ*^2^ test, corrected *χ*^2^ test, or Fisher's exact probability method. Multivariate ANOVA was applied to analyze the factors affecting rehabilitation potential. A *p*-value of <0.05 was considered statistically significant.

## 3 Results

**Figure 1:**
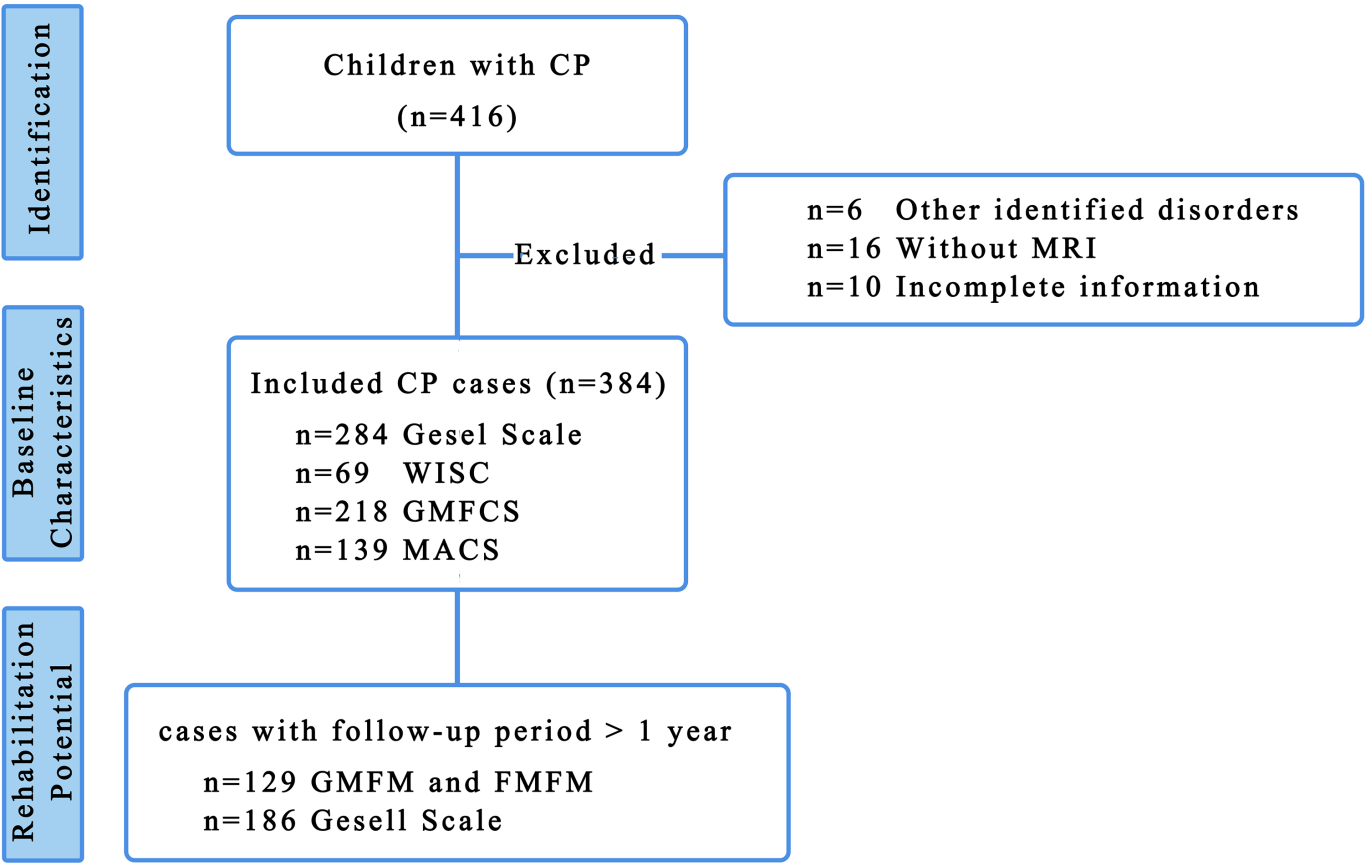
Flow chart of inclusion and exclusion.

In total, 416 children with CP were initially enrolled in the study, with 384 children retained for cross-sectional analysis of clinical characteristics after excluding six cases with alternative diagnosed disorders, 16 cases with incomplete MRI data, and 10 cases with missing information (Figure 1). The median follow-up duration for evaluating rehabilitation potential was 20 months (IQR: 12, 36.5) using GMFM/FMFM and 27 months (IQR: 11.75, 47.25) using Gesell scale.

### 3.1 Epidemiological data

Among the 384 cases analyzed, the male-to-female ratio was 2.34 and the median age of diagnosis was 6.5 months (IQR 4–12). Preterm infants accounted for nearly half of all cases (*n* = 166, 43.2%), with post-term infants accounting for only five cases (1.3%). Spastic CP was the most common phenotype (*n* = 283, 73.6%), including 173 cases with diplegia, 90 cases with hemiplegia, 20 cases with tetraplegia. The mixed type was observed in 21.6% (*n* = 83) of cases. Hypoxic-ischemic encephalopathy (*n* = 93, 24.2%) was identified as the leading cause of CP, followed by brain injuries in preterm infants (*n* = 79, 20.6%). Of the 218 children evaluated by GMFCS, 77 (35.3%) were classified as GMFCS IV-V, while the remainder were classified as GMFCS I–III.

Predominant white matter injury was the most prevalent MRICS categorization (*n* = 156, 40.6%), followed by miscellaneous (*n* = 112, 29.2%), predominant gray matter injury (*n* = 60, 15.6%), maldevelopment (*n* = 21, 5.5%), and normal (*n* = 35, 9.1%) (Table 1).

**Table 1:** The relationship between MRICS, pregnancy history and clinical characteristics.

	MRICS					Total	*p*-value
	Maldevelopments	Predominant white matter injury	Predominant grey matter injury	Miscellaneous	Normal		
	21 (5.5%)	156 (40.6%)	60 (15.6%)	112 (29.2%)	35 (9.1%)	384 (100.0%)	
Sex							
Male	16 (76.2%)	121 (77.6%)	37 (61.7%)	79 (70.5%)	16 (45.7%)	269 (70.1%)	0.002
Female	5 (23.8%)	35 (22.4%)	23 (38.3%)	33 (29.5%)	19 (54.3%)	115 (29.9%)	
The age of diagnosis (m)							
M (IQR)	6 (3–12)	8 (5–12.75)	6 (3–7.75)	6 (3–8.75)	8 (6–12)	6.5 (4–12)	0.001
Gestational age (wk)							
M (IQR)	37 (32.5–38.7)	33 (30.8–37)	39.1 (37.6–40)	38.6 (36–39.7)	38 (36–39.7)	37 (32.4–39)	0.000
≥37+	13 (61.9%)	42 (26.9%)	53 (88.3%)	85 (75.9%)	25 (71.4%)	218 (56.8%)	
28–37	6 (28.6%)	89 (57.1%)	5 (8.3%)	26 (23.2%)	6 (17.1%)	132 (34.4%)	0.000
<28	2 (9.5%)	25 (16.0%)	2 (3.3%)	1 (0.9%)	4 (11.4%)	34 (8.9%)	
Birth weight (kg)							
M (IQR)	2.9 (1.7–3.2)	2 (1.7–2.6)	3.2 (2.9–3.5)	3.1 (2.7–3.4)	3 (2.5–3.3)	2.8 (1.9–3.3)	0.000
≥2.5	12 (57.1%)	50 (32.1%)	54 (90.0%)	89 (79.5%)	27 (77.1%)	232 (60.4%)	
1.5–2.5	7 (33.3%)	86 (55.1%)	6 (10.0%)	15 (13.4%)	5 (14.3%)	119 (31.0%)	0.000
<1.5	2 (9.5%)	20 (12.8%)	–	8 (7.1%)	3 (8.6%)	33 (8.6%）	
CP type							
Spastic	16 (76.2%)	137 (87.8%)	28 (46.7%)	79 (70.5%)	23 (65.7%)	283 (73.7%)	0.000
Hemiplegia	4 (19.0%)	36 (23.1%)	19 (31.7%)	30 (26.8%)	1 (2.9%)	90 (23.4%)	0.000
Diplegia	11 (52.4%)	93 (59.6%)	8 (13.3%)	41 (36.6%)	20 (57.1%)	173 (45.1%)	0.000
Tetraplegia	1 (4.8%)	8 (5.1%)	1 (1.7%)	8 (7.1%)	2 (5.7%)	20 (5.2%)	0.706
Dyskinetic	1 (4.8%)	–	10 (16.7%)	5 (4.5%)	1 (2.9%)	17 (4.4%)	0.000
Ataxia	–	1 (0.6%)	–	–	–	–	–
Mixed	4 (19.0%)	18 (11.5%)	22 (36.7%)	28 (25.0%)	11 (31.4%)	83 (21.6%)	0.000
GMFCS							
Level I–III	7 (50.0%)	68 (76.4%)	22 (71.0%)	29 (50.9%)	8 (57.1%)	134 (65.4%)	0.013
Level IV–V	7 (50.0%)	21 (23.6%)	9 (29.0%)	28 (49.1%)	5 (42.9%)	71 (34.6%)	
Etiologies							
Preterm brain injury	4 (19.0%)	55 (35.3%)	1 (1.7%)	14 (14.3%)	5 (14.3%)	79 (20.6%)	0.000
Hypoxic-ischemic encephalopathy	2 (9.5%)	28 (17.9%)	19 (31.7%)	36 (32.1%)	8 (22.9%)	93 (24.2%)	0.022
Hyperbilirubinemia	1 (4.8%)	1 (0.6%)	12 (20.0%)	3 (2.7%)	3 (8.6%)	20 (5.2%)	0.000
Genetic disorders	–	–	–	3 (2.7%)	2 (5.7%)	5 (1.3%)	–
Craniocerebral trauma	–	–	1 (1.7%)	3 (2.7%)	–	4 (1.0%)	–
Meningitis	–	–	–	4 (3.6%)	–	4 (1.0%)	–
Hypoglycaemia	–	2 (1.3%)	–	1 (0.9%)	–	3 (0.8%)	–

Based on the Kruskal-Wallis and *χ*^2^ tests, significant differences were evident across MRICS groups in terms of sex (*χ*^2 ^= 16.479, *p = *0.002), age of diagnosis (*H* = 19.709, *p = *0.002), gestational age (*H* = 69.792, *p < *0.000), birth weight (*H* = 86.122, *p < *0.000), CP type (*χ*^2 ^= 40.464, *p < *0.000), and GMFCS (*χ*^2 ^= 12.438, *p = *0.013). Predominant white matter injury was associated with male dominance, later diagnosis, lower gestational age, and lower birthweight.

Regarding the CP type, mixed type (36.7%) and spastic hemiplegia were more common in predominant gray matter injury group. In contrast, diplegia was more common in the predominant white matter injury category (59.6%) and in cases with normal MRI results (57.1%).

The distribution of GMFCS scores within MRICS categories exhibited significant variance (*χ*^2 ^= 12.384, *p *= 0.013). Specifically, within the GMFCS scores of I–III, the predominant white matter injury group constituted the majority (71%). In contrast, within the GMFCS scores of IV–V, maldevelopment and miscellaneous categories presented elevated occurrences of 50% and 49.1%, respectively.

### 3.2 Baseline of neurodevelopmental characteristics

#### 3.2.1 Gesell scale

**Figure 2:**
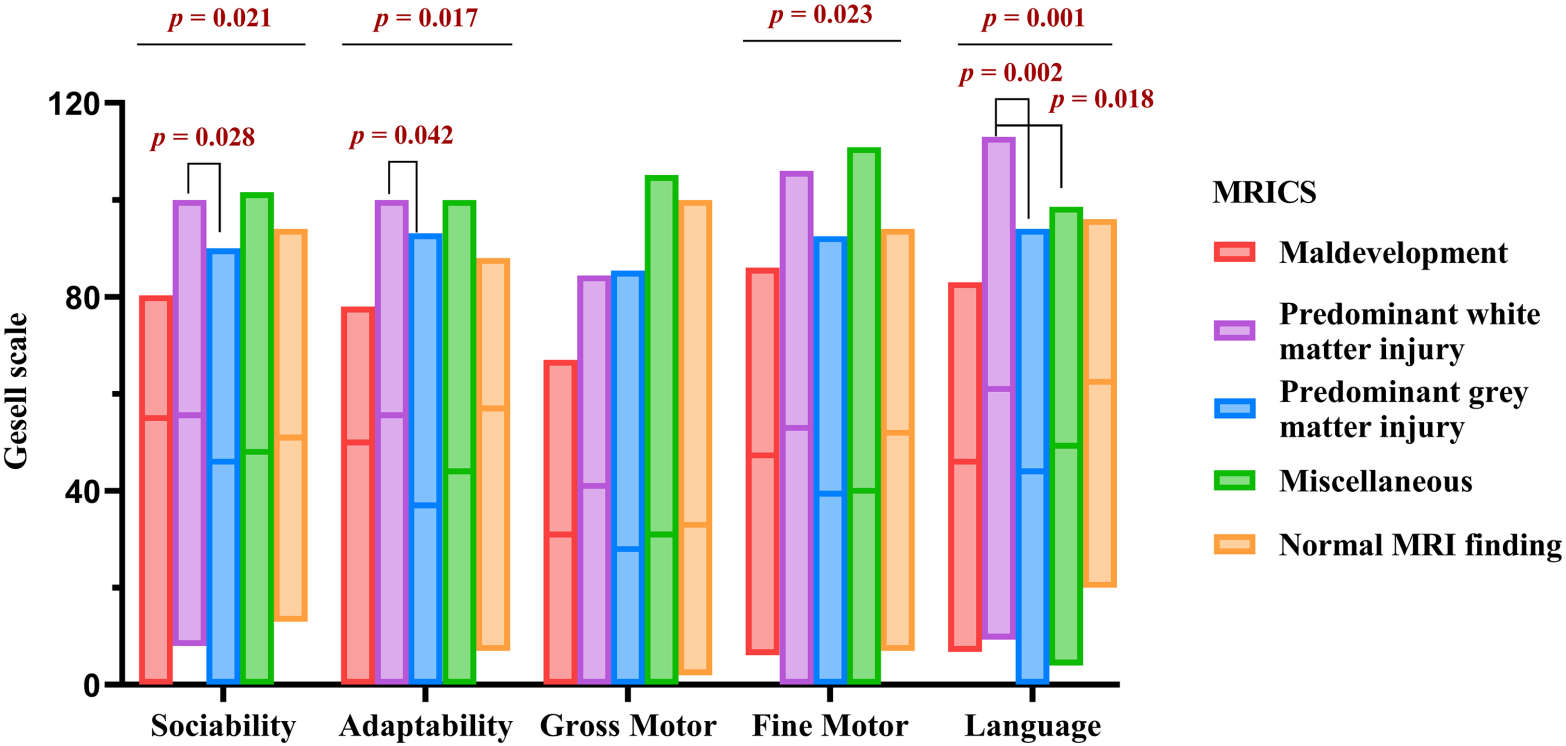
The initial Gesell score of different types of MRICS.

Figure 2 presents the Gesell scale results for 284 children prior to rehabilitation. Significant variations were observed among the MRICS groups in sociability (*H* = 11.559, *p = *0.021), adaptability (*H* = 12.072, *p = *0.017), fine motor ability (*H* = 11.345, *p = *0.023), and language ability (*H* = 19.211, *p = *0.001). Notably, the predominant white matter injury group outperformed the predominant gray matter injury group in terms of sociability (*H* = 41.054, *p = *0.028), language (*H* = 39.407, *p = *0.042), and adaptability (*H* = 51.389, *p = *0.002). Furthermore, language proficiency of the miscellaneous group was inferior to that of the predominant white matter injury group (*H* = 37.112, *p = *0.018).

#### 3.2.2 WISC

The WISC outcomes for 69 children prior to rehabilitation are shown in Supplementary Figure S1. No significant differences were found among the MRICS groups. Nonetheless, a notable decline in IQ performance was associated with age (*p = *0.008), suggesting that the discrepancy between children with CP and their healthy counterparts may expand in the absence of rehabilitative interventions.

#### 3.2.3 GMFCS and MACS

Of the children with predominant white matter injury, 76.4% were classified as GMFCS I–III, notably less severe compared to other groups (*χ*^2 ^= 12.438, *p *= 0.013). Conversely, no significant variation was observed across MRICS categories concerning MACS (*H* = 8.176, *p *= 0.085).

### 3.3 Rehabilitation potential across MRICS

The study included 54 cases characterized by predominant white matter injury, 21 cases with predominant gray matter injury, and 54 cases categorized as miscellaneous. Thus, the rehabilitation potential among these three groups was subsequently analyzed.

#### 3.3.1 GMFM and FMFM

In the examined cohort (*n* = 129), the added GMFM score was 4.3 ± 5.8 in the predominant white matter injury group, 8.5 ± 9.2 in the predominant gray matter injury group, and 6.78 ± 9.54 in the miscellaneous group. For FMFM score was 2.1 ± 5.9 in the predominant white matter injury group, 4.1 ± 7.7 in the predominant gray matter injury group, and 6.5 ± 6.7 in miscellaneous group. Detailed data are provided in Supplementary Figure S2.

Statistical analysis using the Kruskal-Wallis test revealed that gross motor potential measured by GMFM over one year was independent of MRICS (*H* = 1.027, *p = *0.598). Similarly, based on multi-factor analysis, fine motor potential measured by FMFM was not associated with MRICS (*F* = 2.877, *p = *0.060).

#### 3.3.2 Gesell scale

Gesell scale data were obtained for 113 children before and after rehabilitation within the follow-up period, resulting in a total of 186 datasets (Supplementary Figure S3).

Of the five domains evaluated by the Gesell scale, only fine motor ability (*H* = 24.459, *p* < 0.000) and adaptability (*H* = 15.535, *p* = 0.004) showed significant differences in rehabilitation potential across the five MRICS groups over one year, irrespective of age and duration of rehabilitation, as determined by Kruskal-Wallis analysis.

**Figure 3:**
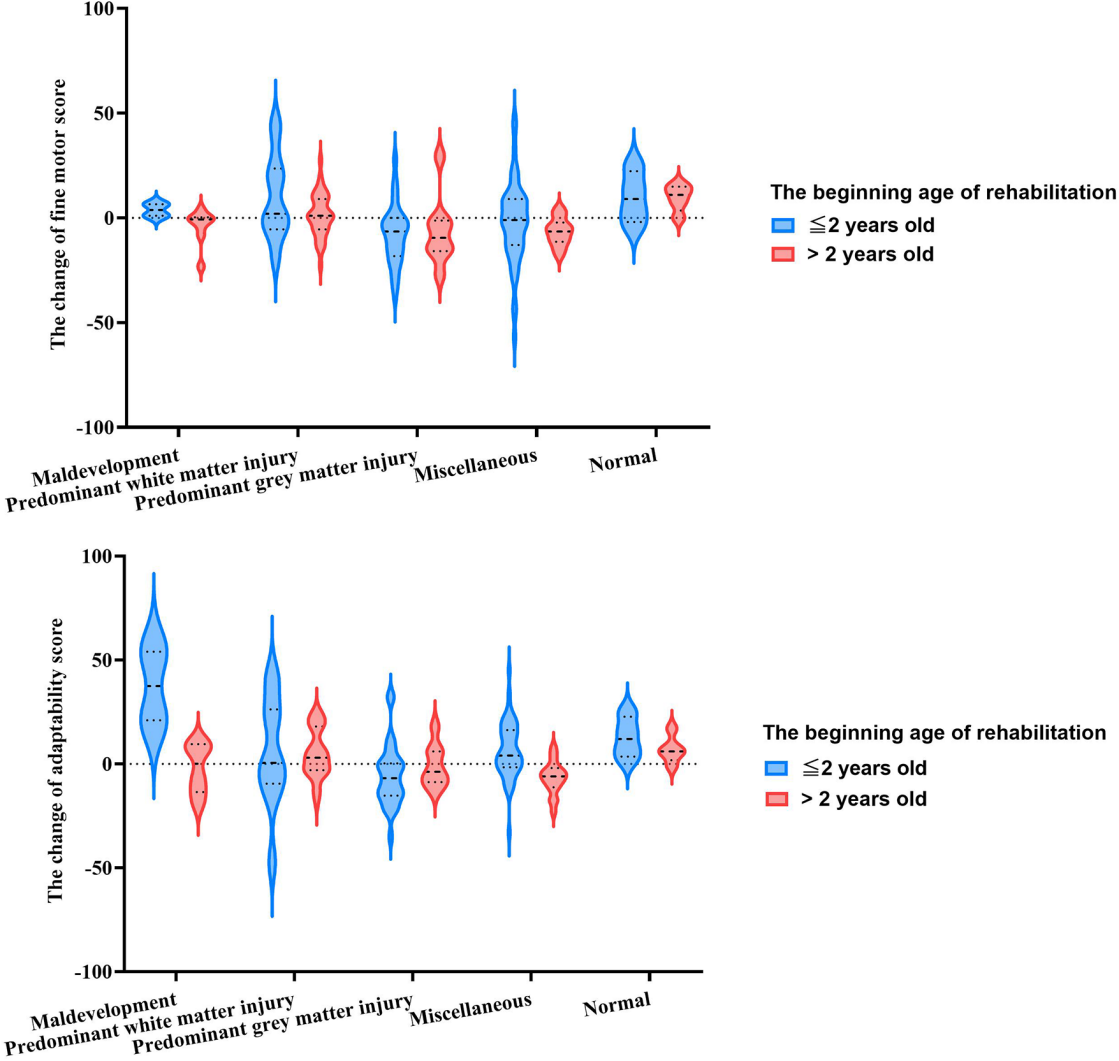
The rehabilitation potential assessed by Gesell scale among MRICS between two onse age groups.

As shown in Figure 3, comparison of rehabilitation potential in fine motor function during the rehabilitation period was in the order: normal > predominant white matter injury > maldevelopment > miscellaneous > predominant gray matter injury (*H* = 24.459, *p < *0.000). This difference in MRICS categories persisted even when age and duration of rehabilitation were included in multivariate analysis (*F* = 6.543, *p < *0.000) (Supplementary Table S1). Rehabilitation potential in children aged ≤2 years was significantly higher than that of children aged >2 years (*F* = 4.015, *p = *0.047). However, no significant difference was observed between the first and second six months of rehabilitation (*F* = 1.910, *p = *0.169).

Similarly, adaptability potential was influenced by MRICS and exhibited a comparable trend (*F* = 4.082, *p = *0.003). The younger age group demonstrated superior rehabilitation potential for adaptability compared to the older age group (*F* = 9.935, *p = *0.002). The variation in rehabilitation potential across MRICS categories varied by age group (*F* = 4.100, *p = *0.003) (Supplementary Table S2). In children younger than 2 years, the maldevelopment group showed the highest potential, followed by normal group (*H* = 18.883, *p = *0.001). In contrast, in those children aged >2 years, the normal group exhibited the most favorable results, followed by the predominant white matter injury group (*H* = 15.314, *p = *0.004). Regarding fine motor function, there was no significant difference between the initial and subsequent six months of rehabilitation in terms of adaptability scores (*F* = 0.259, *p = *0.611).

## 4 Discussion

This research elucidated MRICS-related clinical characteristics, focusing on motor function and neurodevelopmental milestones in children with CP, using a larger sample size than previous studies (6, 7, 14). Predominant white matter injury emerged as the most common MRICS pattern, typically characterized by delayed diagnosis and reduced motor dysfunction relative to other patterns. Importantly, differences in rehabilitation potential, specifically concerning fine motor skills and adaptability, across MRICS may assist health professionals and families in predicting rehabilitation outcomes.Consistent with previous studies (4–7, 15), predominant white matter injury was the most common type and occurred significantly more often in preterm infants. Additionally, a predominant association was detected between hemiplegia spastic and dyskinetic CP and predominant gray matter injury, in accordance with prior research (5–7, 16). This may be attributed to the classification of arterial infarctions as predominant gray matter lesions in MRICS criteria and to the close association between unilateral spastic hemiplegia with perinatal arterial ischemic stroke (6, 17).

As common comorbidities of CP, autism spectrum disorder and attention-deficit/hyperactivity disorder (ADHD) are associated with different regions and networks of brain. PÅhlman et al. found that autism spectrum disorder was frequently associated with predominant white matter injury, while ADHD was more common in predominant grey matter injury group (18). Autism has been found to be associated with the corpus callosum, and the second trimester was a high-risk period for both autism and predominant white matter injury (19). ADHD and predominant grey matter injury, especially cerebral artery infarction, were subject to a later time of injury (20).

As the previous report (4), the cases with predominant white matter injury showed lower percentage of severe motor function by GMFCS. In contrast, Nagy et al. (6) found that cases with normal MRI display lower GMFCS, rather than white matter injury. Some of the apparently normal brain MRI were found to be genetic causes, such as *SPAST*, that presented progressive motor dysfunction over time (21). Thus, the underlying genetic factor and evaluation time of GMFCS may account for the disparities observed among different cohorts. Interestingly, in the current study, individuals with predominant white matter lesions exhibited better sociability, adaptability, fine motor ability, and language ability. While gray matter is involved in information processing centers, white matter establishes connections indicative of neuron communication efficiency (22). In addition, structures such as the basal ganglia and thalamus lack compensatory alternatives in the brain (23). Consequently, the secondary role of white matter in information processing, coupled with its pronounced neuroplasticity, may underpin the milder symptoms observed in cases of predominant white matter injury.

Regarding rehabilitation potential, fine motor ability and adaptability based on the Gesell scale were most pronounced in the normal MRI group, followed by the predominant white matter injury group. Notably, no significant differences were observed across MRICS categories based on the FMFM scale. This discrepancy may stem from the emphasis in the Gesell scale on general developmental functions, such as picking up and grasping small objects, rather than the detailed action of each motion in FMFM. These findings suggest that overall adaptability trajectories pertaining to self-care, community living, communication, and socialization were improved over time (24), while fine motor functions, as assessed by FMFM, were not. In contrast to gross motor skills, fine motor skills are highly correlated with daily living tasks, including cutlery use, dressing, writing, and drawing (25). Fine motor skills are also important for social adaptability and the development of both of them are associated with the cerebellum (26, 27).

Rehabilitation capitalizes on neuroplasticity, with the highest potential observed during the earliest stages of central nervous system development (28, 29). Thus, it is anticipated that the younger cohort would exhibit greater potential compared to the older group. Although rehabilitation potential diminishes over time, extended rehabilitation remains essential (30, 31), particularly for younger children.

This study has several limitations. Firstly, the retrospective nature of this cohort study may introduce information bias, although all data were derived from objective evaluation scales. Secondly, the sample size for comparing rehabilitation potential was relatively small, and not all MRICS patterns were represented. Thirdly, although the at-home and in-hospital rehabilitation protocols were standardized within the same unit, variations in rehabilitation duration, intensity, and familial factors may introduce discrepancies in rehabilitation outcomes, potentially affecting perceived efficacy. Thus, a well-designed prospective cohort study with an appropriate sample size is warranted to elucidate the relationship between MRICS and clinical characteristics as well as rehabilitation potential.

We conclude that the MRICS is closely relevant to the clinical characteristic in Children with CP. Predominant white matter injury ermerged as the most frequent MRICS pattern and was associated with reduced severity. Both normal MRI category andwhite matter injury showed more improved rehabilitation potential in terms of fine motor function and adaptability. Thus, the MRICS may serve as a robust tool for delineating CP clinical attributes and guiding rehabilitation expectations for healthcare professionals and families.

## Data Availability

The raw data supporting the conclusions of this article will be made available by the authors, without undue reservation.
