# Supplementary figures and images for: Clinical characteristics and rehabilitation potential in children with cerebral palsy based on MRI classification system
Source: Front Pediatr. 2024 Apr 25;12:1382172. doi: 10.3389/fped.2024.1382172 (PMC11079180; doi:10.3389/fped.2024.1382172)

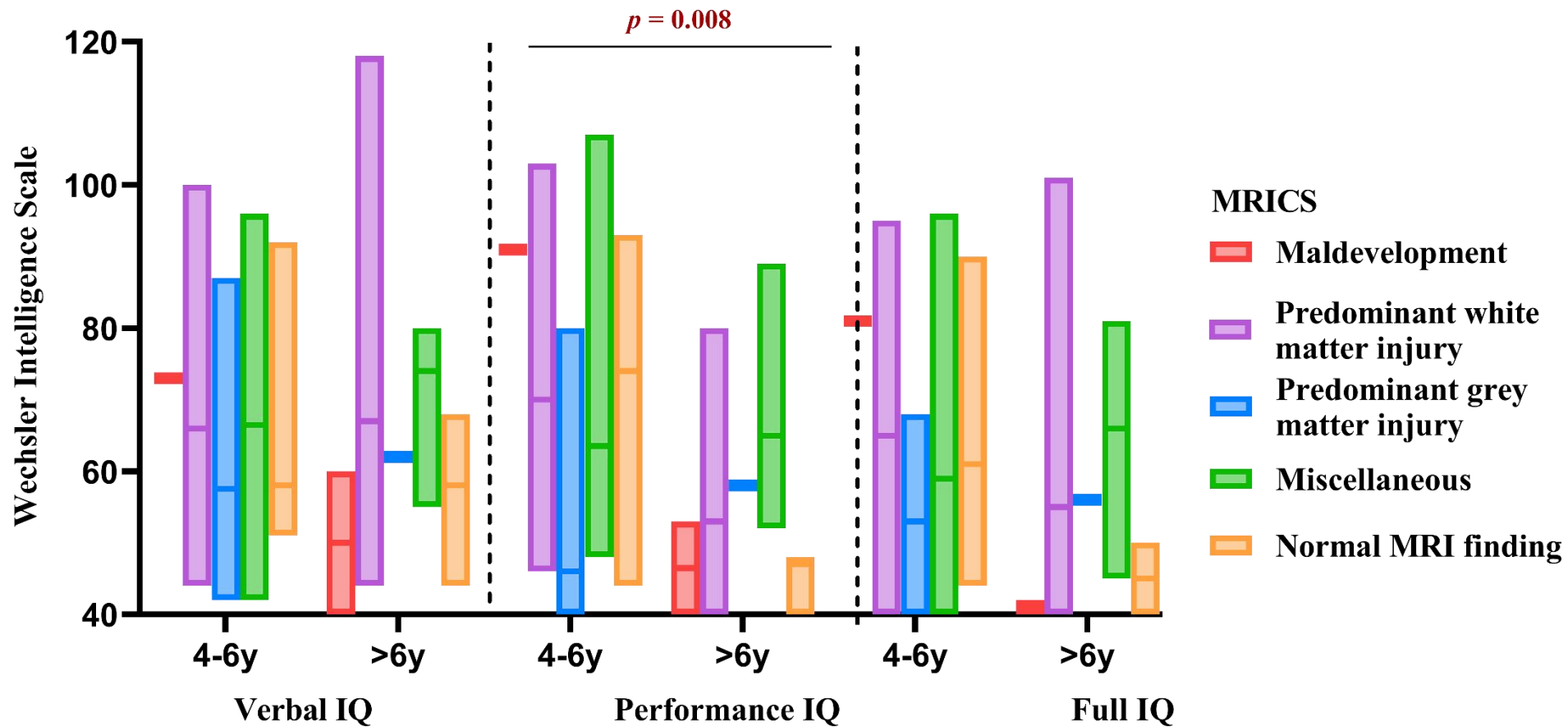

Supplement: Supplementary file 1 [file Image1.pdf]

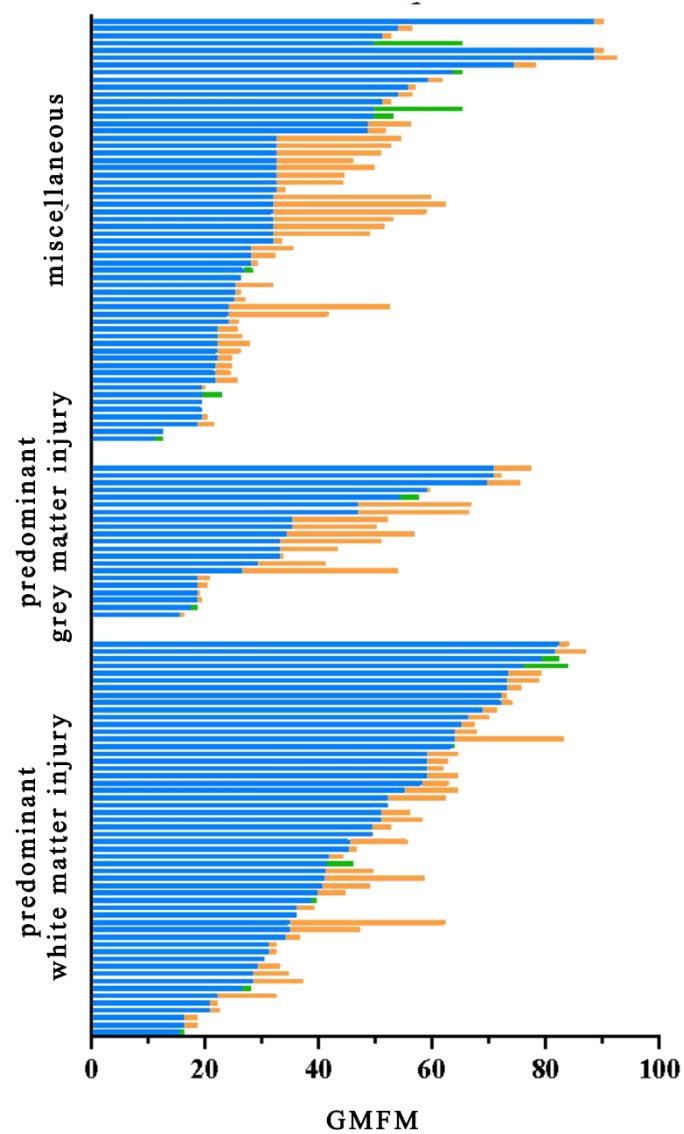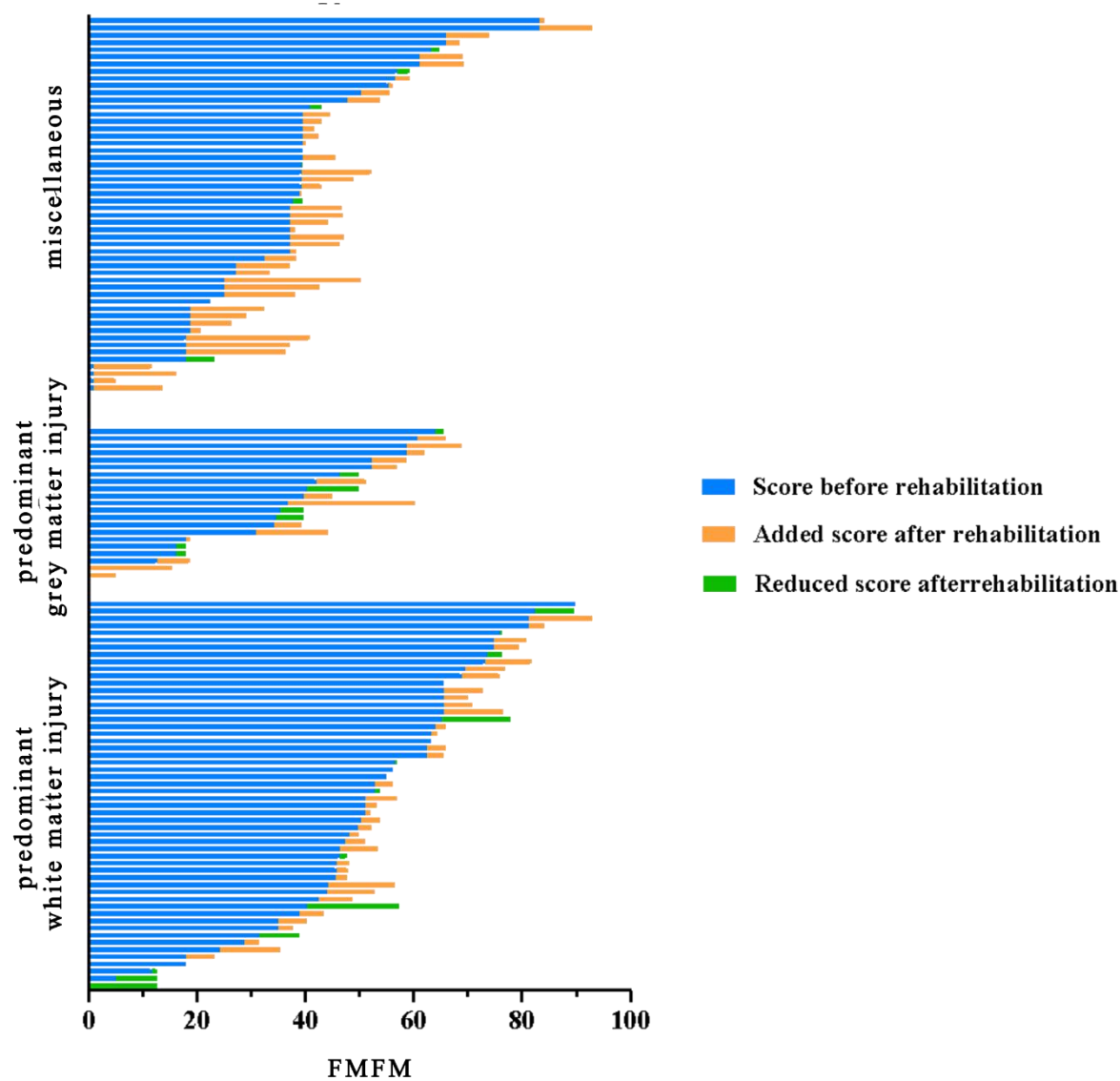

Supplement: Supplementary file 2 [file Image2.pdf]
