# Supplementary material for: Clinical characteristics and rehabilitation potential in children with cerebral palsy based on MRI classification system
Source: Front Pediatr. 2024 Apr 25;12:1382172. doi: 10.3389/fped.2024.1382172 (PMC11079180; doi:10.3389/fped.2024.1382172)
Supplement: Supplementary file 3 [file Image3.pdf]

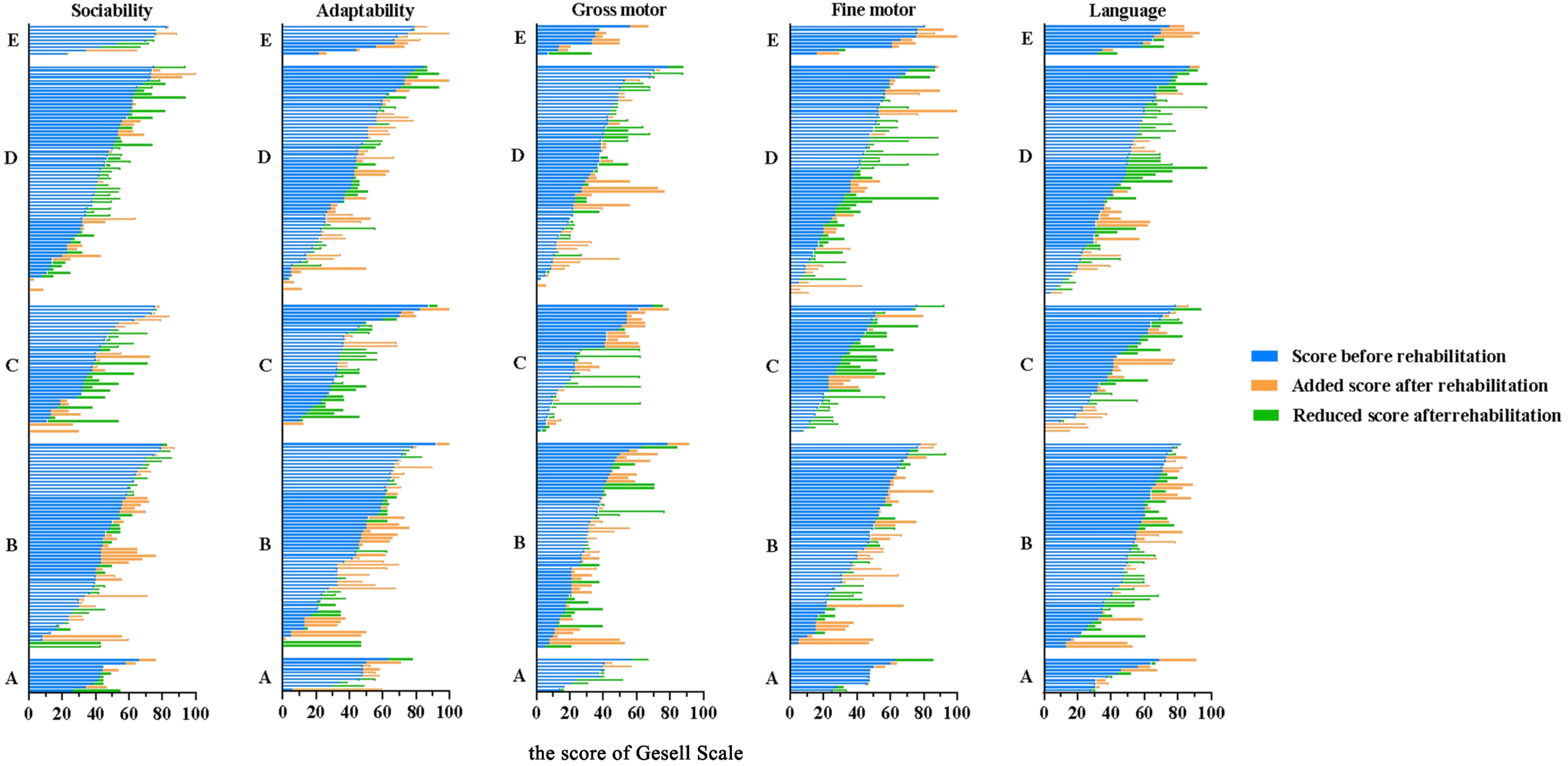

* A.Maldevelopment; B.Predominant white matter injury; C.Predominant grey matter injury; D.miscellaneous; E.Normal finding MRI.
