# Supplementary material for: Clinical characteristics and rehabilitation potential in children with cerebral palsy based on MRI classification system
Source: Front Pediatr. 2024 Apr 25;12:1382172. doi: 10.3389/fped.2024.1382172 (PMC11079180; doi:10.3389/fped.2024.1382172)
Supplement: Supplementary file 4 [file Table1.docx]

| **Table S1.** | The rehabilitation potential of fine motor assessed by the Gesell Scale among MRICS in different age groups. | | | | |
| --- | --- | --- | --- | --- | --- |
| MRICS | | Age | Mean | Standard Deviation | 95% Confidence Interval |
| maldevelopments | | ≦2y | 5.36 | 10.90 | -16.15 ~ 26.87 |
|  |  | >2y | -3.72 | 5.43 | -14.43 ~ 6.98 |
| predominant white matter injury | | ≦2y | 10.58 | 3.28 | 4.11 ~ 17.06 |
|  |  | >2y | 2.01 | 2.51 | -2.94 ~ 6.96 |
| predominant gray matter injury | | ≦2y | -7.59 | 2.81 | -13.13 ~ -2.05 |
|  |  | >2y | -5.95 | 5.43 | -16.66 ~ 4.75 |
| miscellaneous | | ≦2y | -1.90 | 2.17 | -6.19 ~ 2.38 |
|  |  | >2y | -6.59 | 3.61 | -13.72 ~ 0.53 |
| normal | | ≦2y | 10.56 | 7.68 | -4.61 ~ 25.72 |
|  |  | >2y | 10.55 | 6.89 | -3.05 ~ 24.14 |

| **Table S2.** | The rehabilitation potential of adaptability assessed by the Gesell Scale among MRICS in different age groups. | | | | |
| --- | --- | --- | --- | --- | --- |
| MRICS | | Age | Mean | Standard Deviation | 95% Confidence Interval |
| maldevelopments | | ≦2y | 38.08 | 10.57 | 17.23 ~ 58.94 |
|  |  | >2y | -1.91 | 5.26 | -12.29 ~ 8.48 |
| predominant white matter injury | | ≦2y | 5.73 | 3.18 | -0.55 ~ 12.01 |
|  |  | >2y | 5.13 | 2.43 | 0.33 ~ 9.92 |
| predominant gray matter injury | | ≦2y | -5.35 | 2.72 | -10.73 ~ 0.02 |
|  |  | >2y | -0.82 | 5.26 | -11.20 ~ 9.57 |
| miscellaneous | | ≦2y | 6.57 | 2.11 | 2.41 ~ 10.73 |
|  |  | >2y | -6.65 | 3.50 | -13.56 ~ 0.26 |
| normal | | ≦2y | 13.04 | 7.45 | -1.66 ~ 27.75 |
|  | | >2y | 7.27 | 6.68 | -5.91 ~ 20.45 |
